# Splenectomy is significantly associated with thrombosis but not with pulmonary hypertension in patients with transfusion-dependent thalassemia: a meta-analysis of observational studies

**DOI:** 10.3389/fmed.2023.1259785

**Published:** 2023-10-11

**Authors:** Tsampika-Vasileia Kalamara, Konstantinos Dodos, Efthymia Vlachaki

**Affiliations:** ^1^Adults Thalassemia Unit, Hippokration General Hospital, Second Department of Internal Medicine, Aristotle University of Thessaloniki, Thessaloniki, Greece; ^2^Third Department of Pediatrics, Hippokration General Hospital, Aristotle University of Thessaloniki, Thessaloniki, Greece

**Keywords:** thalassemia, splenectomy, thrombosis, pulmonary hypertension, meta-analysis, observational

## Abstract

**Introduction:**

Thromboembolism (TE) and pulmonary hypertension (PH) constitute frequently occurring complications in patients with transfusion-dependent thalassemia and have been associated with splenectomy in different studies. Nevertheless, the size of the possible association varies greatly in literature. Herein, we sought to provide pooled effect estimates regarding the impact of splenectomy on TE and PH in transfusion dependent thalassemia (TDT) by retrieving relevant, available studies.

**Methods:**

We systematically searched articles published in PubMed, Cochrane library, Scopus and gray literature from inception until the 30th of May, 2023. Pooled estimates in terms of odds ratios (OR) and 95% confidence intervals (CI) were calculated according to outcome measures. Risk of bias and quality of studies were evaluated.

**Results:**

Regarding TE, 4 studies were selected for meta-analysis and the pooled data demonstrated that splenectomy was significantly associated with this outcome in TDT patients [OR = 4.08, 95% CI (1.03, 16.11), *p* = 0.04]. On the other hand, we pooled data from seven investigating PH, and, interestingly, the quantitative analysis revealed no association between splenectomy and PH [OR = 1.76, 95% CI (0.91, 3.41), *p* = 0.1].

**Conclusion:**

Splenectomy is associated with higher risks of TE, but not with PH in patients with TDT.

## 1. Introduction

Thalassemia syndromes are a diverse collection of congenital autosomal recessive hemoglobinopathies caused by globin gene abnormalities, most of which are minor nucleotide substitutions. The two primary types of the disease, alpha-thalassemia and beta-thalassemia, are distinguished by decreased or absent synthesis of either the alpha-or beta-globin chains of the hemoglobin molecule (1). Cooley and Lee described thalassemias for the first time almost a century ago, when they reported cases of severe anemia with splenomegaly and distinctive bone changes (2). At least one variant globin allele is carried by an estimated 5% of the global population, with a higher prevalence observed in the Mediterranean region, the Middle East, Africa, Southeast Asia, and the Indian subcontinent (1).

Patients with beta-thalassemia have a variety of clinical manifestations. Historically, this illness was classified as thalassemia major, intermedia, and minor based on the globin chain ratio (alpha/beta), degree of anemia, and clinical history. Asymptomatic microcytic anemia is common in patients with beta-thalassemia minor (carrier or trait). Thalassemia syndromes are nowadays classified into two groups depending on clinical severity and transfusion requirement: TDT and Non-Transfusion-Dependent Thalassemia (NTDT). TDT patients are unable to produce enough hemoglobin to survive without transfusions. Transfusions are rarely required in NTDT patients (3). TDT necessitates maintaining a pre-transfusion hemoglobin level of 95–105 g/L, which suppresses erythropoiesis and allows for a reduction in blood consumption. The higher standards for pretransfusional hemoglobin levels that current transfusion guidelines in TDT are setting are usually attained by more frequent transfusions (4).

Despite advances in therapeutic care in the era of novel drugs such as luspaterecpt, splenectomy remains an essential treatment option in TDT patients, particularly in low-income countries, and is considered in the following cases: higher transfusion needs (200–220 mL of red blood cells/kg/year), symptomatic splenomegaly, signs of hypersplenism resulting in clinical problems (4). The therapeutic rationale for splenectomy, particularly in patients suffering from poor health due to thalassemia-induced medical conditions, is to protect against the establishment of extramedullary hematopoiesis by increasing hemoglobin levels, decreasing the need for transfusions, and, as an ultimate result, minimizing iron overload (5). Both open and laparoscopic techniques are employed for total splenectomy, with the latter requiring shorter hospitalization and appearing to offer lower morbidity and short-term mortality. In a limited number of centers, partial splenectomy, which preserves some immune functions of the spleen, as well as embolization of splenic tissue are evaluated, although not widely accepted. Adverse events following splenectomy include bleeding, atelectasis, subphrenic abscess, extreme thrombocytosis and overwhelming post-splenectomy sepsis. Morbidities are common in people with TDT. The procedure of splenectomy has been related to major long-term complications including thromboembolic and PH consequences, as well as infections, which increase morbidity and death risk in these patients (4). The spleen normally removes damaged red cells. In the absence of spleen, high levels of negatively charged RBCs, as well as high levels of platelets which present with hyperactivity, seem to contribute to the hypercoagulable state of thalassemia, as these cell elements stay in the blood circulation and activate thrombin production mechanisms (6).

PH is most frequently identified in NTDT, although it is also becoming more common in TDT lately. This complication is diagnosed and monitored by echocardiography (tricuspid valve jet velocity), while cardiac catheterization is often used for validation. Pulmonary vasodilator therapies are used for the management of PH. Additionally, thalassemia patients tend to present with an increased risk for arterial and venous thrombosis. Risk factor education with regard to the avoidance of other risk factors, aspirin prophylaxis for at-risk individuals and routine anticoagulation are included in the therapeutic management of TE in TDT patients. Nevertheless, TDT is a lifelong high-burden disease both for patients and for healthcare systems (3). The scientific community must invest in better understanding the etiology of the disease complications and the elements that influence its natural history, since this will lead the development of new therapeutics, as well as appropriate and timely use of the already available agents.

Therefore, we sought to determine the impact of splenectomy on two major complications, namely TE and PH, by performing the first in the literature systematic review and meta-analysis of relevant studies.

## 2. Methods

This systematic review and meta-analysis is conducted in accordance with the PRISMA (Preferred Reporting Items for Systematic reviews and Meta-Analyses) guidelines (7) and the Meta-analysis Of Observational Studies in Epidemiology (MOOSE) guidelines (8).

### 2.1. Eligibility criteria

We searched the articles published in PubMed, Cochrane library, Scopus and gray literature from inception until the 30th of May, 2023. We searched for studies enrolling adult patients diagnosed with TDT who were splenectomized compared to non-splenectomized TDT patients, reporting on the complications TE and PH. We did not impose any restriction regarding language of publication, study design (retrospective, prospective), setting and sample size. We excluded case reports, case series, former meta-analyses (if any), editorial and opinion papers, narrative reviews.

### 2.2. Search strategy

We searched articles published in PubMed, Cochrane library, Scopus, and gray literature, namely conference proceedings, including full-text articles in English. We did not impose any filter regarding sample size, study setting, or publication language. MeSH terms were used for both intervention and outcomes, along with free-text words. We also used the Boolean operators “OR” and “AND.” The searching strategy applied in PubMed is shown in Supplementary Appendix Table 1.

### 2.3. Data extraction

Following deduplication, two independent reviewers (T-VK, KD) screened all records at title and abstract level and then assessed the full text of eligible records. Any disagreements were resolved by consultation of a third reviewer (EV).

Two independent reviewers (T-VK, KD) extracted the data from the eligible reports. Relevant information was extracted and recorded on a data collection form developed in Microsoft Excel^©^. Extracted information included the following: first author, year of study conduction, country of origin, study sample size, key clinical outcomes (TEE, PH), measurement method of PH, type of thrombosis.

The Newcastle-Ottawa Scale (NOS) (9) was used by two independent reviewers (T-VK, KD) to assess the quality of the included observational studies. The included studies were evaluated based on three general criteria: study participants, group comparability, and determination of either the exposure or outcomes of interest. Any individual study can receive up to four stars for selection, two stars for comparability, and three stars for outcome, with a maximum score of nine stars. Divergent views among reviewers were settled through debate, consensus, or arbitration by a third senior reviewer (EV).

### 2.4. Data synthesis and analysis

We planned to assess major clinical endpoints (TE, PH) representing dichotomous variables, thus the OR with 95% CI were estimated. To generate the pooled estimates of the outcomes, the Mantel–Haenszel (M-H) random effects formula was implemented. To assess the extent to which statistical heterogeneity in meta-analysis is due to differences between studies rather than accidental, we used I^2^ statistic. Heterogeneity was considered to be low if I^2^ was between 0 and 25%, moderate if I^2^ was between 25 and 50%, or high if I^2^ was greater than 75% (10). The forest plots were used for a visual representation of the presence and nature of statistical heterogeneity. A value of *p* < 0.05 was considered statistically significant. All statistical analyses were performed using the RevMan 5.3. software (11, 12).

## 3. Results

### 3.1. Data sources and selection process

**Figure 1:**
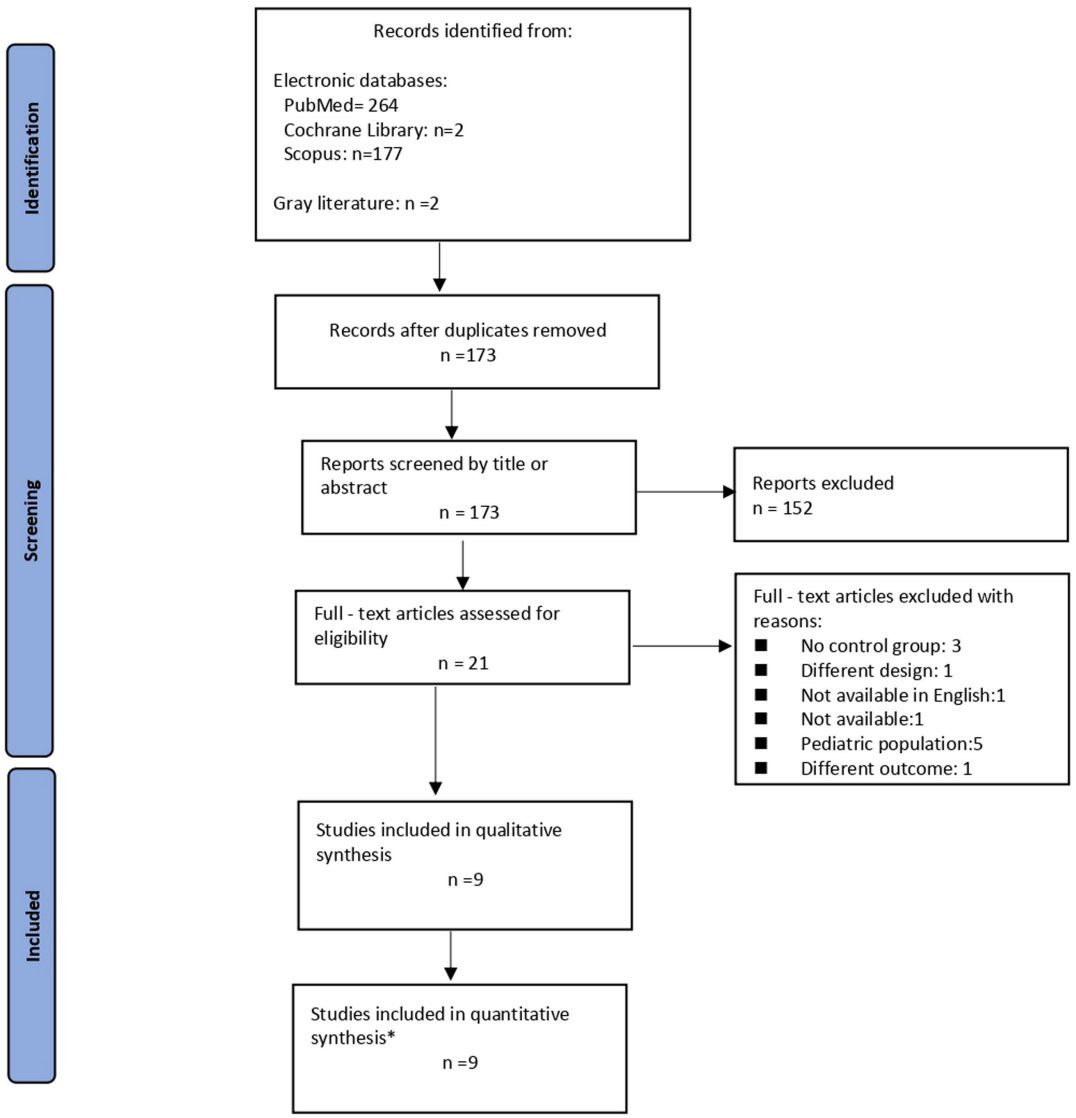
Flow diagram illustrating the study selection process. *Two studies were common fo the two outcomes (TE, PH) and were used in both analyses.

As shown in the corresponding PRISMA flow diagram (Figure 1), our search strategy retrieved 445 results in total. After deduplication, we initially screened 173 records at title and abstract level. Finally, we assessed 21 records in full text. Nine (13–21) of them were evaluated as eligible for inclusion in our qualitative synthesis. Of these, four studies were included in the quantitative synthesis regarding the outcome TE and seven studies were used in the final PH meta-analysis. Twelve observational studies were excluded for various reasons (Figure 1). Five studies (22–26) were excluded from the analysis due to different population (pediatric population). Moreover, three studies (27–29) were not included due to the lack of control group. The results presented by another study were also excluded, due to the fact that a different outcome was assessed (quality of life) (30). The study of Alieva et al. (31) was not included because the full study was only available in Russian. Additionally, the study by Derchi et al. (32) was not taken into account due to study design differences. Finally, the study by Taher and colleagues (33) could not be included, as original data were not available after contact with the author.

### 3.2. Characteristics of the included studies

A detailed description of participants’ baseline characteristics is provided in Table 1. Regarding the primary outcome TE, we pooled data from four studies in a total of 690 enrolled subjects. Overall, 15 cases of thrombosis were detected, of which 14 (93%) were observed on splenectomized patients. Thromboembolic events included portal vein thrombosis (PVT), deep vein thrombosis (DVT), pulmonary embolism (PE), transient ischemic attack (TIA) and cerebrovascular disease. The commonest adverse event was PVT (47%), followed by DVT (27%). Most events were recorded between 1 and 5 years after splenectomy, while the patients were receiving low dose aspirin (80-100 mg). As far as the outcome PH is concerned, data were collected from 7 studies, with a total of 395 participants. Sixty-three patients were referred with PH, 45 (71%) of whom were splenectomized.

**Table 1:** Baseline characteristics of the included studies.

Study	Splenectomized/non-splenectomized	Country	Outcome	Outcome assessment	Follow-up	Results
Cappellini (13)	48/17	Italy	TE	U/S, color doppler/venography, V/Q scan, angiography, CT, MRI	10 years	S group: 1 transient ischemic attack NS group: 0
Esfahani (14)	31/29	Iran	PH	echocardiography	–	S group: 4 NS group: 0
Hagar (15)	17/11	USA	PH	echocardiography	–	S group: 10 NS group: 6
Hassan (16)	160/160	Iran	TE	color Doppler ultrasound	11 years	S group: 5 PVT(1 month to 3 years after splenectomy) NS group: 0
Kalamara (17)	73/68	Greece	TEE PH	U/S, color doppler/venography, v/q scan, angiography, CT echocardiography	25.75 ± 11.7 years	■ TEE S group: 4 (DVTx3, PVT x1), NS group: 1 DVT ■ PH S group: 7 NS group: 3
Meera (18)	6/9	India	PH	echocardiography	–	S group: 2 NS group: 1
Meloni (19)	24/36	China, Southeast Asia, Indian Subcontinent, Italy, Greece, Cyprus, Middle East	PH	echocardiography	21 months	S group: 1 NS group: 0
Morsy (20)	36/15	Saudi Arabia	PH	echocardiography	–	S group: 14 NS group: 6
Osataphan (21)	TE: 44/20 PH: 27/12	Thailand	TEE PH	echocardiography	5 years	■ TEE S group: 4 (cerebrovascular disease x2, pulmonary embolism x1, PVT x1) NS group: 0 ■ PH S group: 7 NS group: 2

### 3.3. Meta-analysis

**Figure 2:**
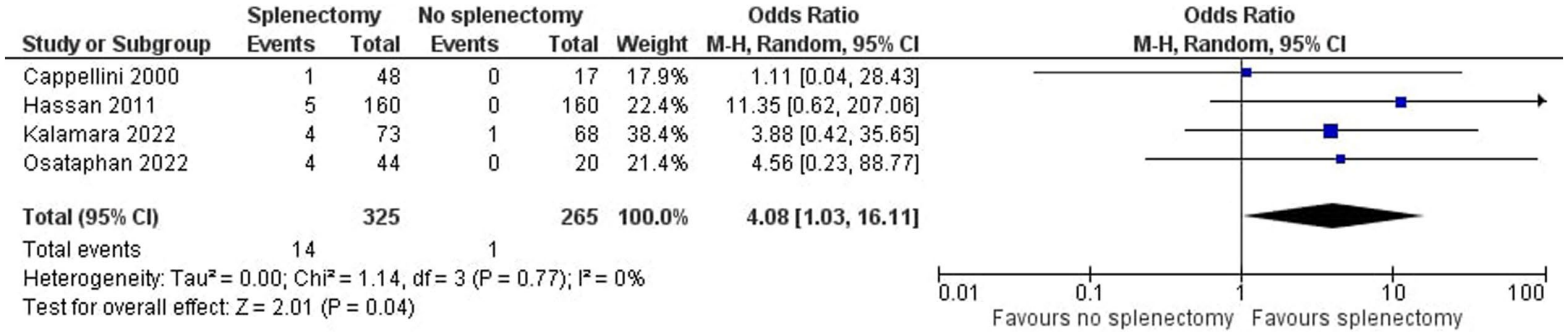
Odds ratio for thromboembolism.

**Figure 3:**
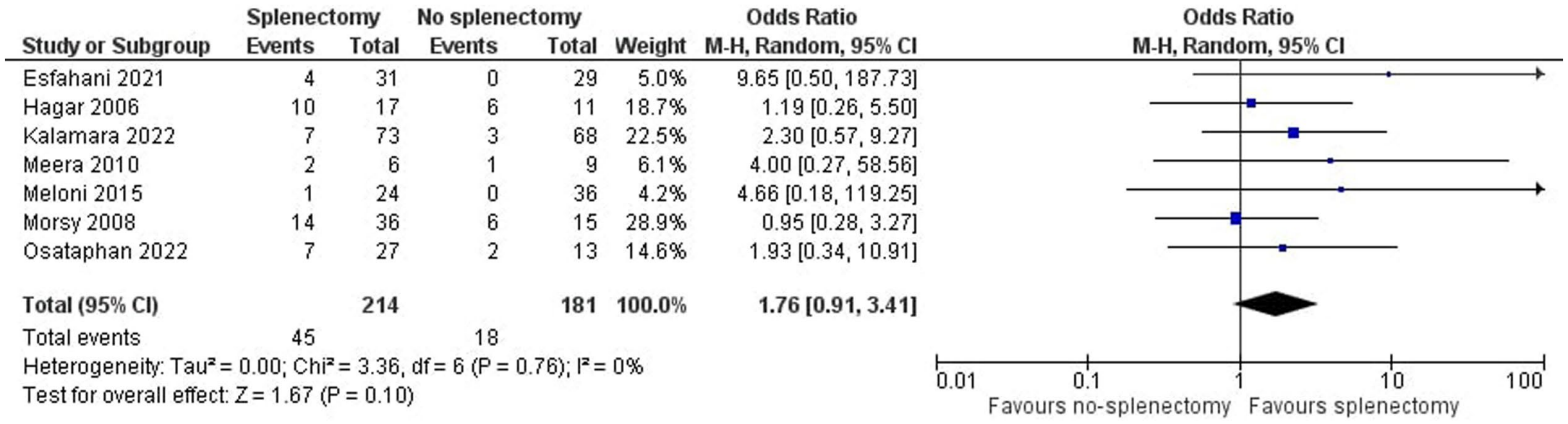
Odds ratio for pulmonary hypertension.

As shown in Figure 2, we demonstrated that splenectomy is associated with a statistically significant higher prevalence of TE in TDT patients [OR = 4.08, 95% CI (1.03, 16.11)], with the test for overall effect conforming statistical significance (*p* = 0.04). Notably, no association between splenectomy and PH was proved [OR = 1.76, 95% CI (0.91, 3.41), *p* = 0.10] according to Figure 3. Heterogeneity of the included studies was low in both analyses (I^2^ = 0).

### 3.4. Assessment on quality of studies

The risk of bias appraised among the included studies is shown in Table 2. Study quality scores ranged from 6 to 9, and all of them were of good quality.

**Table 2:** Newcastle-Ottawa quality assessment form regarding included studies.

Studies	Representative exposed cohort	Selection of non-exposed cohort	Ascertainment of exposure	Precision of Exposure Dose Ascertainment	Outcome of interest not present at baseline	Adjustment for confounding	Outcome assessment	Was follow-up long enough for outcomes to occur?	Adequacy of follow-up	Quality
Cappellini (13)	⋆	⋆	⋆	⋆	⋆	–	⋆	⋆	⋆	good
Esfahani (14)	⋆	⋆	⋆	⋆	⋆	–	⋆	–	⋆	good
Hagar (15)	⋆	⋆	⋆	⋆	⋆	–	⋆	–	–	good
Hassan (16)	⋆	⋆	⋆	⋆	⋆	–	⋆	⋆	⋆	good
Kalamara (17)	⋆	⋆	⋆	⋆	⋆	–	⋆	⋆	⋆	good
Meera (18)	⋆	⋆	⋆	⋆	⋆	–	⋆	–	–	good
Meloni (19)	⋆	⋆	⋆	⋆	–	–	⋆	⋆	–	good
Morsy (20)	⋆	⋆	⋆	⋆	⋆	–	⋆	–	–	good
Osataphan (21)	⋆	⋆	⋆	⋆	⋆	–	⋆	⋆	⋆	good

## 4. Discussion

In this study we attempted to synthesize and assess the already documented evidence, as derived by the existing observational studies, regarding the risk of splenectomized thalassemic patients in developing TE or PH. In order for this to be achieved, a systematic review of the already existing bibliography was conducted according to the PRISMA guidelines (7). Thus, we utilized meta-analysis as a robust tool and followed an organized approach, in order to assimilate data and combine results from multiple independent studies answering our research question.

According to our results, statistical significance (*p* = 0.04 < 0.05) was observed with regard to the outcome TE, but no evidence of significant association was found regarding PH (*p* = 0.10 > 0.05). Our conclusions may be less definite, as some of the comparisons in the included studies arise from retrospective data and historical controls, while, additionally, a retrospective design often lacks full availability of risk factors data and comorbidities. Specifically, both thrombosis and PH are rather complicated and multifactorial phenomena, so a direct causative relation between these events and splenectomy in TDT patients is difficult to be unraveled. Nevertheless, our results should be highlighted, although they must be confirmed in large-scale prospective, well-designed trials in the future.

Certain studies suggest that there is still a lot of evidence that splenectomy has negative effects on both healthy people and people with hematological diseases, such as TDT. The most frequently documented and concerning complications are increased susceptibility to infections and TE. However, a significant portion of thalassemic patients will continue to undergo splenectomy until a substitute is advised by evidence-based guidelines. These patients, along with those who have already undergone splenectomy, constitute a substantial group of patients who are at a possible risk for splenectomy-related complications.

TDT patients may benefit from splenectomy in cases that, despite advantageous chelation therapy, iron overload in not sufficiently reduced, leading to life-threatening excess iron deposits mainly in the liver, heart, and endocrine organs. For these patients, splenectomy, in the context of a comprehensive management of iron overload, is efficient in reducing the rate of transfusional iron loading. Moreover, this procedure is valuable in erasing the symptoms of early satiety and left upper quadrant pain, as well as the risk of a possible splenic rupture, in patients experiencing massive splenomegaly, thus, improving quality of life and reducing the morbidity and mortality risk. Last but not least, thalassemia patients who experience clinical conditions such as bleeding or recurrent infections, as a result of hypersplenism causing thrombocytopenia and leucopenia respectively, may overcome these adverse events through splenectomy.

However, splenectomy has been related to many disadvantages and unpleasant conditions. Except for PH and hypercoagulation, which are more thoroughly discussed in the present work, infections constitute a sizable long-term risk. Overwhelming sepsis in splenectomized TDT patients is commonly related to encapsulated pathogens such as *Streptococcus pneumoniae* (75% of cases), *Haemophilus influenzae* and *Neisseria meningitidis*, gram-negative organisms and protozoa, with a greater risk being documented after 1–4 years following the procedure. Prevention of overwhelming sepsis is the most important measure to avoid this complication. Proper education should be offered to patients, so that they have the ability to recognize febrile illnesses and report them to their physician. Moreover, pneumococcal, *Haemophilus influenzae* and meningococcal polysaccharide vaccine should be properly and timely administered to thalassemic patients undergoing splenectomy for the achievement of immunoprophylaxis. Finally, children under 5 years of age should be treated with prophylactic antibiotics, such as chemoprophylaxis with oral penicillin (4, 6).

Many studies have shown that increased red blood cells and platelet count were associated not only with higher risk of TE events, but also with shorter time between splenectomy and TE. Red-cell senescence antigens, such phosphatidylserine and membrane proteins undergo iron-dependent oxidation in hemolytic anemia, which in turn causes thalassemic RBCs to be stiff, distorted, and aggregate, leading to premature cell removal (28, 33–41). Phospholipids with a negative charge may be present in thalassemic RBCs, which may eventually lead to an increase in the production of thrombin (42, 43). Splenectomized patients had considerably more circulating RBCs with negatively charged phospholipids (44). Additionally, such patients had, also, considerably higher amounts of circulating RBC microparticles (submicrometric membrane fragments with procoagulant potential) compared to controls (45). After a blood transfusion the quantity of circulating damaged RBCs is reduced (46). These results may help to partially explain why patients with high nuclated RBC counts or transfusion-naive patients experienced more TE episodes. A few observational studies have also shown that thalassemic patients who receive blood transfusions present less often with TEE, PH, and silent brain infarcts than transfusion-naive patients (47, 48). Correction of the underlying inefficient erythropoiesis and the consequent damaged RBCs with thrombogenic potential may be responsible for this. As a result, early transfusion therapy that aims to prevent the effects of chronic hemolytic anemia may assist patients by preventing such issues rather than only treating them after they have already occurred and are irreversible. It is, also, of note, that in the recent years, several studies have linked transfusions of red blood cells to thromboembolism (49), in the context of a variety of illnesses affecting both inpatients and outpatients. Lin and colleagues in an observational retrospective study with over 41,000 participants found that blood transfusions were significantly associated with venous thromboembolism, but, also, that with the administration of warfarin this risk was decreased (50). So, anticoagulation issues, as well as a possible thrombophilic predisposition should be always taken into consideration by physicians.

Patients with thalassemia and hereditary stomatocytosis were the first to suggest a connection between splenectomy and PH. According to estimates, it takes a significant amount of time (between two and 35 years) after splenectomy for PH to manifest (13, 51–53). In 58 thalassemia patients who had undergone splenectomy, 54% had pulmonary vascular alterations suggestive of microthromboemboli, as opposed to 16% of the remaining patients (13, 42, 44–59). The PH in asplenic patients with thalassemia is typically categorized as chronic thromboembolic pulmonary hypertension, which typically affects the distal pulmonary arteries and has a distinctive histopathology (42, 55). It is also likely that the process is actually “*in situ*” thrombosis, which is characterized by medial hypertrophy, intimal fibrosis, and plexiform lesions and is associated with idiopathic PH (55). Increased cardiac output as a result of chronic anemia, decreased plasma concentration of antithrombotic agents such as protein C, S and Antithrombin III could lead to platelet activation and microthrombotic episodes leading to RBC membrane malformations (57). The asymmetric RBC membrane phospholipids (56, 57), nitric oxide scavenging by free hemoglobin, and subsequent endothelial dysfunction brought on by nitric oxide depletion are some putative pathophysiologic mechanisms of PH in patients with hemolytic anemia. Those highly thrombogenic red blood cells would normally be eliminated by splenic macrophages, but, in the absence of this organ, they tend to be present in blood circulation for much longer and can increase hypercoagulability (58, 59).

Vasoconstriction expressed by abnormal narrowing of the pulmonary arteries is a primary mechanism in pulmonary hypertension. This constriction can be due to imbalances in the production of vasoconstrictors such as endothelin-1 or deficiencies in vasodilators like nitric oxide (NO) (60). In turn, increased vasoconstriction leads to elevated resistance and pressure in the pulmonary arteries. That, in addition to dysfunction of the endothelial cells, lining the pulmonary arteries, is commonly observed in pulmonary hypertension (60, 61). Endothelial cells play a crucial role in maintaining vascular tone and regulating blood flow. The remodeling of pulmonary arteries is, also, a hallmark of pulmonary hypertension. It involves structural changes in the arterial walls, including smooth muscle cell proliferation, fibrosis, and the formation of plexiform lesions (62). These changes lead to the narrowing of the vessel lumen, further increasing pulmonary vascular resistance. Another pathophysiologic mechanism involves Inflammation and immune dysregulation. Immune cells, such as macrophages and lymphocytes, infiltrate the pulmonary arteries, releasing pro-inflammatory cytokines and growth factors that promote vascular remodeling and vasoconstriction (60, 61). As far as genetic and hereditary factors are concerned, they can also be related with certain forms of pulmonary hypertension. Mutations in genes involved in signaling pathways, such as bone morphogenetic protein receptor type 2, can disrupt normal vascular homeostasis and contribute to the development of pulmonary hypertension (60). Lastly, pulmonary vascular thrombosis can contribute to the development or exacerbation of PH (62). Someone can safely state that the pathophysiology of PH in splenectomized thalassemic patients is a rather complicated phenomenon with a large number of contributing factors. The extent of the effect of each factor cannot be measured according to the already existing literature, so further studies should be conducted in the direction of further explaining this rather difficult circumstance.

As far as NTDT is concerned, there is a decline in the use of splenectomy over the last years, as this procedure has been linked to several important negative outcomes. Similarly to TDT, hypercoagulability issues arise from the removal of the spleen, as procoagulant RBCs, erythroblasts and platelets, and probably iron free fractions, cannot be scavenged. NTDT patients present with an increased (reaching 5-fold) risk of venous TE, PH, silent cerebral infarction and leg ulcers, compared to unsplenectomized patients. These results arise from a number of previous studies, although, to our knowledge, no synthesis of the available data in the form of a meta-analysis has been conducted and published. Pulmonary hypertension in NTDT patients is characterized by increased pressure in pre-capillary pulmonary vessels. Probable causes have been identified and it seems that the exact pathophysiology of the phenomenon is rather complex. Chronic thromboembolic disease, chronic anemia/hypoxia and the subsequent hyperdynamic circulation as well as disruptions in the synthetic pathways of vasodilators such as nitrous oxide derivatives can be blamed as probable causes of this clinical syndrome (63). Thus, a probable explanation of the fact that PH was not associated with splenectomy in TDT patients according to our results, but has been observed in NTDT population according to numerous studies, could be the chronic hemolytic anemia and the subsequent hyperdynamic circulation derived by chronic hypoxia, which in TDT may be alleviated by recurrent transfusions.

In accordance with our study results, that showed an association between splenectomy and the serious adverse event of TE, we would not recommend splenectomy as a standard-of care measure. Patients should be advised to undergo splenectomy only in cases of extreme transfusion requirements and clinical conditions that make this procedure inevitable. We should emphasize on availability of optimal transfusion regimens and strict transfusion protocols and chelation treatment, which minimize the incidence of splenomegaly and reduce iron overload. In cases that patients are already splenectomized, careful monitoring is of great significance. Awareness of the possible risks is required. TE events and infections should be immediately addressed and patients should be well informed and trained to recognize these conditions. In cases that splenectomy must be conducted, laparoscopic procedure seems to be a safer option, and precise immunization and chemoprophylaxis protocols should be followed. Low dose aspirin should be prescribed to all post-splenectomy patients, unless this is contraindicated. In cases that other risk factors for thrombosis are present, low molecular weight heparin prophylaxis should also be considered.

To our knowledge, this is the first meta-analysis and systematic evaluation of the relationship between splenectomy and TEE and PH in TDT. Our study has certain advantages. We established the link between splenectomy and thrombosis through evidence synthesis, and the finding may have important clinical implications for in the clinical setting. Moreover, we showed that splenectomy had a neutral effect on PH in these patients, despite the traditional views on this subject. Additionally, we created appropriate inclusion and exclusion criteria, resulting in a data collection that is rather homogeneous, according to the heterogeneity testing. The inclusion of quality assessment was also a great tool, allowing readers to appraise the level of evidence. Finally, two independent reviewers completed the research and data extraction, which enables us to confirm the review’s comprehensiveness and accuracy. Nevertheless, an important limitation to be mentioned is that, despite the high link between splenectomy and thrombosis, causality could not be fully established, as some of the included studies were retrospective, cross-sectional or case–control studies. Thus, more prospective well-designed cohort studies are needed for this result to be confirmed in the future.

## Data availability statement

The original contributions presented in the study are included in the article/Supplementary material, further inquiries can be directed to the corresponding author.

## Author contributions

T-VK: Writing – original draft, Writing – review & editing. KD: Writing – original draft, Writing – review & editing. EV: Writing – review & editing.
