# Supplementary material for: Splenectomy is significantly associated with thrombosis but not with pulmonary hypertension in patients with transfusion-dependent thalassemia: a meta-analysis of observational studies
Source: Front Med (Lausanne). 2023 Oct 11;10:1259785. doi: 10.3389/fmed.2023.1259785 (PMC10598854; doi:10.3389/fmed.2023.1259785)
Supplement: Supplementary file 1 [file Table_1.docx]

| **PubMed (up to 30/05/2023)** | | |
| --- | --- | --- |
| **Search** | Query | Items found |
| **#1** | Search “Thalassemia” [Mesh] | 24824 |
| **#2** | Search thalassemia [All Fields] | [31871](https://pubmed.ncbi.nlm.nih.gov/?term=thalassemia&sort=&filter=dates.1000/1/1-2023/5/30) |
| **#3** | Search transfusion-dependent thalassemia [All Fields] | [1653](https://pubmed.ncbi.nlm.nih.gov/?term=transfusion-dependent+thalassemia&sort=&filter=dates.1000/1/1-2023/5/30) |
| **#4** | Search TDT [All Fields] | [7623](https://pubmed.ncbi.nlm.nih.gov/?term=TDT&sort=&filter=dates.1000/1/1-2023/5/30) |
| **#5** | Search beta-thalassemia [All Fields] | [17039](https://pubmed.ncbi.nlm.nih.gov/?term=beta-thalassemia&sort=&filter=dates.1000/1/1-2023/5/30) |
| **#6** | Search #1 OR #2 OR #3 OR #4 OR #5 | [39252](https://pubmed.ncbi.nlm.nih.gov/?term=((((Thalassemia%5bMeSH+Terms%5d+AND+(1000/1/1:2023/5/30%5bpdat%5d))+OR+(thalassemia+AND+(1000/1/1:2023/5/30%5bpdat%5d)))+OR+(transfusion-dependent+thalassemia+AND+(1000/1/1:2023/5/30%5bpdat%5d)))+OR+(TDT+AND+(1000/1/1:2023/5/30%5bpdat%5d)))+OR+(beta-thalassemia+AND+(1000/1/1:2023/5/30%5bpdat%5d))&sort=&filter=dates.1000/1/1-2023/5/30) |
| **#7** | Search “Thromboembolism” [Mesh] | [64142](https://pubmed.ncbi.nlm.nih.gov/?term=Thromboembolism%5bMeSH+Terms%5d&sort=&filter=dates.1000/1/1-2023/5/30) |
| **#8** | Search thromboembolism [All Fields] | [112033](https://pubmed.ncbi.nlm.nih.gov/?term=thromboembolism&sort=&filter=dates.1000/1/1-2023/5/30) |
| **#9** | Search thrombosis [All Fields] | 267006 |
| **#10** | Search embolism [All Fields] | 193979 |
| **#11** | Search #7 OR #8 OR #9 OR #10 | 458193 |
| **#12** | Search “Pulmonary hypertension” [Mesh] | [41892](https://pubmed.ncbi.nlm.nih.gov/?term=Pulmonary+hypertension%5bMeSH+Terms%5d&sort=&filter=dates.1000/1/1-2023/5/30) |
| **#13** | Search pulmonary hypertension [All Fields] | [79517](https://pubmed.ncbi.nlm.nih.gov/?term=Pulmonary+hypertension&sort=&filter=dates.1000/1/1-2023/5/30) |
| **#14** | Search #12 OR #13 | 79517 |
| **#15** | Search “Splenectomy” [Mesh] | 22557 |
| **#16** | Search splenectomy [All Fields] | 32051 |
| **#17** | Search splenectomized [All Fields] | 3730 |
| **#18** | Search splenectomised [All Fields] | 412 |
| **#19** | Search #15 OR #16 OR #17 OR #18 | 33404 |
| **#20** | Search #11 OR 14 | 527517 |
| **#21** | Search #6 AND #19 AND # 20 | 264 |
